# Biological pathways underlying the association of red cell distribution width and adverse clinical outcome: Results of a prospective cohort study

**DOI:** 10.1371/journal.pone.0191280

**Published:** 2018-01-17

**Authors:** Giedre Zurauskaite, Marc Meier, Alaadin Voegeli, Daniel Koch, Sebastian Haubitz, Alexander Kutz, Luca Bernasconi, Andreas Huber, Mario Bargetzi, Beat Mueller, Philipp Schuetz

**Affiliations:** 1 Medical University Department, Kantonsspital Aarau, Aarau, Switzerland; 2 Institute of Laboratory Medicine, Kantonsspital Aarau, Aarau, Switzerland; 3 Division of Hematology, Oncology and Transfusion Medicine, Kantonsspital Aarau, Aarau, Switzerland; New York University School of Medicine, UNITED STATES

## Abstract

**Background:**

Red cell distribution width (RDW) predicts disease outcome in several patient populations, but its prognostic value in addition to other disease parameters in unselected medical inpatients remains unclear. Our aim was to investigate the association of admission RDW levels and mortality adjusted for several disease pathways in unselected medical patients from a previous multicenter study.

**Methods:**

We included consecutive adult, medical patients at the time point of hospital admission through the emergency department into this observational, cohort study. The primary endpoint was mortality at 30-day. To study association of admission RDW and outcomes, we calculated regression analysis with step-wise inclusion of clinical and laboratory parameters from different biological pathways.

**Results:**

The 30-day mortality of the 4273 included patients was 5.6% and increased from 1.4% to 14.3% from the lowest to the highest RDW quartile. There was a strong association of RDW and mortality in unadjusted analysis (OR 1.32; 95%CI 1.27–1.39, p<0.001). RDW was strongly correlated with different pathways including inflammation (coefficient of determination (R^2^) 0.30; p<0.001), nutrition (R^2^ 0.20; p<0.001) and blood diseases (R^2^ 0.30; p<0.001 The association was eliminated after including different biological pathways into the models with the fully adjusted regression model showing an OR of 1.02 (95%CI 0.93–1.12; p = 0.664) for the association of RDW and mortality. Similar effects were found for other outcomes including intensive care unit admission and hospital readmission.

**Conclusion:**

Our data suggests that RDW is a strong surrogate marker of mortality in unselected medical inpatients with most of the prognostic information being explained by other disease factors. The strong correlation of RDW and different biological pathways such as chronic inflammation, malnutrition and blood disease suggest that RDW may be viewed as an unspecific and general “chronic disease prognostic marker”.

## Introduction

The red cell distribution width (RDW) is a measure of the variability in size within the red cell population [1], which is traditionally known as anisocytosis. Normally it is measured automatically as part of the complete blood count (CBC). For a long time, RDW has been mainly used for the differential diagnosis of anemias.

In recent years different studies and meta-analysis have shown that RDW has an independent predictive ability for mortality in various conditions, i.e. cardiovascular diseases [2], heart failure [3], non-cardiovascular emergencies [4], and cancer [5]. Furthermore RDW was associated with poor prognosis in broader spectrum of diseases including venous thrombosis [6] and community-acquired pneumonia [7]. RDW was also found to predict mortality in older adults [8] and the general population [9]. Different studies have also shown that additional RDW inclusion in disease severity scores increases their predictive value, i.e. SAPS [10], HEART [11], SIRS, MEDS and CURB65 [12]. Yet, in most of these studies, the biological mechanisms explaining this association was not further explored. Due to the lack of rigorous adjustment of statistical regression models in some reports, it remained unclear whether RDW was indeed an independent outcome predictor or rather a marker mirroring other diseases. In addition, only few studies have investigated the prognostic significance of RDW in unselected and thus more general medical inpatients and patients from the emergency department (ED). One study investigating RDW in a general inpatient population was limited by not reporting detailed data on comorbidities and other blood markers. [13]

Herein, we evaluated the association of RDW and mortality as well as other outcomes in a large, unselected medical inpatient population from a previous trial. Our aim was to better understand which pathways would explain the prognostic value of RDW and clinical outcomes. This information may help to better understand the value of this inexpensive and widely available laboratory marker in general medical inpatients and different subgroups that could enhance early risk stratification in the future.

## Methods

### Study design

In this observational cohort study, which was performed as substudy of TIRAGE study, we included consecutive mostly medical inpatients presenting with a medical urgency between March 2013 and October 2014 at a Swiss tertiary care hospital. The institutional Review Board of the Canton Aargau approved the TRIAGE study and waived the need for informed consent, due to the study design (observational quality control study) (EK 2012/059). The initial trial was registered at the “ClinicalTrials.gov” registration website (NCT01768494). All authors had complete access to all the study data, and have reviewed and approved the final manuscript.

### Study population

This study was accomplished in the Department of Medicine in the Kantonsspital Aarau, a 600-bed tertiary care hospital with additional regional primary and secondary care functions with most medical admissions entering the hospital through the Emergency Department (ED). We consecutively included adult patients seeking ED care for medical health issues and in whom an initial blood draw was done as part of the routine ED assessment. Patients hospitalized for surgery as well as children were excluded. Clinical information, including socio-demographic characteristics, main medical diagnosis, comorbidities, and patient outcomes were assessed prospectively until hospital discharge using the routinely gathered information from the hospital electronic medical system used for coding of Diagnosis-Related Group codes. The left-over blood samples collected from all patients upon ED admission were stored at -80°C and then used for later measurement of biomarkers.

All patients were contacted 30 days after hospital admission for a telephone interview with a predefined questionnaire to assess vital and functional status, satisfaction with care provided, hospital readmission and mortality. If a patient could not be reached, family or the general practitioner was contacted to assess vital status. The mortality data was completed with over 98% of patients and/or third person were reached for this assessment. Standardized surveillance methods were not available.

### Laboratory measurements

RDW was conducted as a part of complete blood count on Sysmex XN-9000 (Sysmex Co., Kobe, Japan) automated hematology analyzer. The normal reference range for RDW at our institution was < 15%.

Aside from regularly used biomarkers, blood markers from three different distinct biologic pathways were measured. We took a marker for inflammation (pro-adrenomedulin [ProADM]), stress (copeptin) and bacterial infection (Procalcitonine [PCT]). ProADM, copeptin and PCT were batch-measured in plasma with a highly sensitive time-resolved amplified cryptate emission (TRACE) technology assay (MR-proADM, PCT Kryptor®, B.R.A.H.M.S. AG, Hennigsdorf, Germany). The assays have analytical detection limits of 0.05 nmol/L, 0.9 pmol/L and 0.02 μg/l, respectively.

To describe characteristics of the overall population we used RDW quartiles, which in our population referred to the following ranges: 11%– 12.6%, 12.7% - 13.2%, 13.3% - 14.4%, 14.5% - 30.9%.

For subgroup analysis we divided overall population into 2 groups: with anemia or without anemia. We assigned patients according to their hemoglobin level, where hemoglobin < 120 g/l for men and women were considered to be cut off for anemia.

### Definitions of main diagnosis and comorbidities

Main diagnosis was grouped according to the International Classification of Diseases (10th Revision) (ICD-10). The system altogether contains 22 groups for different conditions. Since some of the groups in our study were left with having a very small amount of patients, we clustered them under “Others”, which included: factors influencing health status, external causes, puerperium complications/other obstetric conditions, diseases of the genitourinary system, diseases of the skin and diseases of the ear and eye. For further analysis, we focused on the following comorbidities coded based on the medical record: chronic obstructive pulmonary disease (COPD), dementia, diabetes mellitus, hypertension, coronary heart disease, stroke, renal failure and tumor.

## Primary and secondary endpoints

The primary outcome of interest of this study was all-cause 30-day mortality. Secondary outcomes were unplanned admission to Intensive Care Unit (ICU) and 30-day readmission rate. All outcomes were ascertained during the hospital stay and at day 30 (after hospital admission) through structured phone interviews done by blinded study nurses.

## Statistical analysis

Descriptive statistics of demographic and laboratory variables are expressed as counts/percentages and continuous variables as medians (interquartile ranges [IQR], 25^th^ and 75^th^ percentiles). The χ^2^ and ANOVA tests were performed for patient characteristics and comparisons between groups. We first used univariate regression analysis to investigate the possible factors to influence the variability in RDW (estimation of coefficient of determination with the adjusted R). Based on those results we implemented stepwise analysis where variables with the highest adjusted R took place first. For the stepwise analysis, as well as multivariate and logistic regressions we used multiple imputation for all missing values as recommended. Sensitivity analysis limited to patients with no missing data was performed first and showed similar results to our main analysis. When entering RDW within quartiles into the models showed similar results compared to the main analysis with RDW as a linear predictor.

The distribution of the copeptin, ProADM, PCT, C-reactive protein (CRP), creatinine, leukocyte count, mean corpuscular volume (MCV), glucose, corrected calcium were skewed. After logarithmic transformation with a base of 10, the distribution of the biomarker data approximated a normal distribution for copeptin, pro-adrenomedullin, PCT, CRP, glucose. To reach normal distribution for creatinine, corrected calcium and leukocyte count, we used a square root and for MCV a square transformation.

We also used logistic models with odds ratios (OD) and relative 95% confidence intervals (95% CI) for binary endpoints. Association of RDW and outcomes were assessed in the overall population and within different predefined subgroups based on laboratory and blood count findings as well as comorbidities. We also ran a multivariate analysis to test different subgroups effect on RDW. Four statistical models were used to adjust for possible confounding: model 1 for age and gender; model 2 age, gender and comorbidities; model 3: age, gender, comorbidities and main diagnosis; model 4: age, gender and laboratory parameters. Full model: age, gender, main diagnosis, comorbidities and laboratory findings.

Additionally, we developed statistical models for different biological pathways: *Cardiovascular system*: RDW, diseases of the circulatory system, comorbidities: hypertension, coronary heart disease; *Lungs*: RDW, diseases of the respiratory system, comorbidity: chronic obstructive pulmonary disease; *Kidneys*: RDW, comorbidity: renal failure, creatinine; *Tumor*: RDW, neoplastic diseases, comorbidity: tumor*; Blood*: RDW, INR, Platelets count, hemoglobin, mean corpuscular volume, diseases of the blood and blood-forming organs; *Nutrition*: RDW, glucose, calcium, calcium corrected, endocrine and metabolic diseases, comorbidity: diabetes; *Inflammation*: RDW, pro-adrenomedullin, copeptin, procalcitonin, C-reactive protein, albumin, leukocyte count, absolute neutrophil count, infectious and parasitic diseases.

All statistical analyses were performed with STATA 12.1 (Stata Corp, College Station, TX, USA), p < 0.05 (two-tailed) was considered statistically significant.

## Results

### Characteristics of the population

We included 4273 medical patients upon hospital admission. The median age of patients was 63 years and 44.2% were female. The largest group of the patients had diseases of the circulatory system (24.6%) as a main diagnosis and many patients suffered from hypertension (42.1%) as a comorbidity. Other common comorbidities were: diabetes (15.0%), renal failure (15.5%) and tumor (14.8%). Laboratory findings and other specifications of the population are presented in **Table 1.**

**Table 1:** Characteristics of the study population.

		*RDW Quartiles*				
Characteristics	Overall	Quartile 1 (11%– 12.6%)	Quartile 2 (12.7% - 13.2%)	Quartile 3 (13.3% - 14.4%)	Quartile 4 (14.5% - 30.9%)	p value
	N = 4273	N = 1183	N = 978	N = 1051	N = 1061	
***Demographic characteristics***						
Age median, median (IQR)	63 (47, 76)	49 (32, 64)	62 (46, 75)	68 (55, 79)	71 (60, 79)	**<0.001**
Female, No. (%)	1781 (44.2%)	498 (44.7%)	399 (43.2%)	433 (44.1%)	451 (44.7%)	0.91
***Main diagnosis***						**<0.001**
Infectious and parasitic diseases, No. (%)	405 (9.6%)	100 (8.7%)	83 (8.7%)	94 (9.0%)	128 (12.1%)	
Neoplastic diseases, No. (%)	231 (5.5%)	28 (2.4%)	22 (2.3%)	45 (4.3%)	136 (12.9%)	
Diseases of the blood^†^, No. (%)	407 (9.7%)	156 (13.5%)	100 (10.4%)	84 (8.1%)	67 (6.3%)	
Endocrine and metabolic diseases, No. (%)	67 (1.6%)	21 (1.8%)	11 (1.1%)	12 (1.2%)	23 (2.2%)	** **
Mental and behavioral diseases, No. (%)	168 (4.0%)	60 (5.2%)	45 (4.7%)	39 (3.8%)	24 (2.3%)	
Diseases of the nervous system, No. (%)	393 (9.3%)	144 (12.5%)	97 (10.1%)	112 (10.8%)	40 (3.8%)	
Diseases of the circulatory system, No. (%)	1034 (24.6%)	231 (20.1%)	280 (29.2%)	278 (26.7%)	245 (23.2%)	
Diseases of the respiratory system, No. (%)	258 (6.1%)	53 (4.6%)	42 (4.4%)	77 (7.4%)	86 (8.1%)	*** ***
Diseases of the digestive system, No. (%)	258 (6.1%)	32 (2.8%)	31 (3.2%)	48 (4.6%)	118 (11.2%)	
Diseases of the MS system^‡^, No. (%)	229 (5.4%)	51 (4.4%)	40 (4.2%)	44 (4.2%)	36 (3.4%)	** **
Symptoms and signs*, No. (%)	501 (11.9%)	181 (15.7%)	130 (13.6%)	117 (11.3%)	73 (6.9%)	
Injury, poisoning, No. (%)	133 (3.2%)	50 (4.3%)	31 (3.2%)	31 (3.0%)	21 (2.0%)	
Other, No. (%)	209 (5.0%)	45 (3.9%)	46 (4.8%)	59 (5.7%)	59 (5.6%)	** **
***Comorbidities***						** **
COPD, No. (%)	216 (5.1%)	25 (2.1%)	27 (2.8%)	70 (6.7%)	94 (8.9%)	**<0.001**
Dementia, No. (%)	138 (3.2%)	17 (1.4%)	27 (2.8%)	50 (4.8%)	44 (4.1%)	**<0.001**
Diabetes, No. (%)	643 (15.0%)	106 (9.0%)	131 (13.4%)	165 (15.7%)	241 (22.7%)	**<0.001**
Hypertension, No. (%)	1799 (42.1%)	327 (27.6%)	382 (39.1%)	547 (52.0%)	543 (51.2%)	**<0.001**
Coronary heart disease, No (%)	536 (12.5%)	109 (9.2%)	135 (13.8%)	143 (13.6%)	149 (14.0%)	**<0.001**
Stroke, No. (%)	429 (10.0%)	84 (7.1%)	128 (13.1%)	114 (10.8%)	103 (9.7%)	**<0.001**
Renal failure, No. (%)	661 (15.5%)	58 (4.9%)	85 (8.7%)	193 (18.4%)	325 (30.6%)	**<0.001**
Tumor, No. (%)	632 (14.8%)	80 (6.8%)	78 (8.0%)	141 (13.4%)	333 (31.4%)	**<0.001**
***Inflammation/infection***						
Pro-adrenomedullin nmol/l, median (IQR)	0.83 (0.61, 1.29)	0.62 (0.5, 0.79)	0.74 (0.58, 1.0)	0.92 (0.69, 1.32)	1.41 (0.97, 2.29)	**<0.001**
Albumin g/l, median (IQR)	37.5 (33.7, 40.6)	39.6 (36.8, 42.3)	38.7 (36, 41.4)	37 (33.8, 39.7)	33.3 (28.6, 37.1)	**<0.001**
CRP mg/l, median (IQR)	5.8 (0, 29.6)	0 (0, 12.1)	3.4 (0, 12.3)	6.55 (0, 29.6)	21.3 (5.7, 82.1)	**<0.001**
PCT μg/l, median (IQR)	0.08 (0.06, 0.13)	0.06 (0.05, 0.09)	0.07 (0.05, 0.1)	0.08 (0.06, 0.13)	0.13 (0.08, 0.29)	**<0.001**
WBC 10^9^/l, median (IQR)	8.49 (6.68, 11.05)	8.18 (6.58, 10.36)	8.34 (6.69, 10.68)	8.56 (6.9, 11.09)	8.99 (6.52, 12.27)	**<0.001**
ANC 10^9^/l, median (IQR)	5.54 (4.09, 7.74)	5.07 (3.84, 7.36)	5.41 (3.97, 7.39)	5.74 (4.31, 7.65)	6.26 (4.51, 8.89)	**<0.001**
***Stress***						
Copeptin pmol/l, median (IQR)	11.9 (4.7, 45.1)	6.65 (3.4, 20.7)	9.6 (4.2, 31.8)	13.5 (5.3, 47.2)	28.1 (9.1, 81.2)	**<0.001**
***Hematological parameters***						
Hemoglobin g/l, median (IQR)	136 (122, 148)	143 (133, 154)	141 (131, 152)	135 (122, 147)	117 (98, 133)	**<0.001**
MCV fl, median (IQR)	89.7 (86.5, 93.2)	89.3 (86.8, 92.1)	89.5 (86.7, 92.7)	90.2 (86.8, 93.9)	89.9 (84.8, 94.5)	**<0.001**
RDW %, median (IQR)	13.2 (12.6, 14.4)	12.2 (12, 12.4)	13 (12.8, 13.2)	13.7 (13.5, 14)	15.8 (15, 17.2)	**<0.001**
Platelets G/l, median (IQR)	233 (189, 286)	232 (195, 275)	235 (195, 279)	232 (188, 286)	235 (170, 308)	0.96
INR, median (IQR)	1.1 (1, 1.2)	1.1 (1, 1.1)	1.1 (1, 1.1)	1.1 (1, 1.2)	1.2 (1.1, 1.6)	**<0.001**
***Renal function***						
Creatinine mmol/l, median (IQR)	86 (71, 107)	79 (67, 93)	84 (70, 100)	89 (72, 110)	98 (77, 138.5)	**<0.001**
***Nutritional status***						
Glucose mmol/l, median (IQR)	6.2 (5.4, 7.5)	5.9 (5.3, 7)	6.1 (5.3, 7.4)	6.3 (5.5, 7.5)	6.5 (5.6, 8.2)	**<0.001**
Calcium mmol/l, median (IQR)	2.2 (2.13, 2.27)	2.22 (2.15, 2.28)	2.21 (2.15, 2.28)	2.2 (2.13, 2.28)	2.18 (2.08, 2.26)	**<0.001**
Calcium corrected mmol/l, median (IQR)	2.26 (2.19, 2.35)	2.23 (2.16, 2.3)	2.25 (2.18, 2.32)	2.27 (2.21, 2.36)	2.34 (2.24, 2.45)	**<0.001**
***Outcome***						
Admission to ICU	175 (4.1%)	35 (3.0%)	35 (3.6%)	44 (4.2%)	61 (5.7%)	**0.007**
30-day readmission	291 (6.8%)	51 (4.3%)	64 (6.5%)	74 (7.0%)	102 (9.6%)	**<0.001**
30-day mortality	238 (5.6%)	16 (1.4%)	25 (2.6%)	44 (4.2%)	152 (14.3%)	**<0.001**

Data are presented as median (IQR) or % (no.). p values are statistically significant at p < 0.05. IQR = Interquartile range (25th-75th percentiles); CRP, C-reactive protein; PCT, procalcitonin; WBC, white blood cell count; ANC, absolute neutrophil count; MCV, mean corpuscular volume; RDW, red cell distribution width; Comorbidities were identified from the hospital electronic medical system.

* Symptoms, signs, abnormal clinical and lab findings

^†^Diseases of the blood and blood-forming organs

^‡^Diseases of the musclosceletal system

### Characteristics of the population according to RDW quartiles

We divided the overall population according to RDW quartiles. Patients in the 4^th^ RDW quartile were significantly older when compared to the 1^st^ quartile (71 vs. 49 years, p < 0.001). They also had more neoplastic (12.9% vs. 2.4%) and digestive (11.2% vs. 2.8%) diseases as main diagnosis. Also, inflammation and stress markers were significantly higher in patients in the the 4^th^ RDW quartile. Regarding clinical outcomes, we found higher risks for all outcomes in the 4^th^ RDW quartile including 30-daymortality, admission to ICU and 30-day readmission. (**Table 1**)

### Primary endpoint

In the overall population, 238 (5.6%) patients reached the primary endpoint of 30-day mortality. More patients with high RDW reached the primary endpoint outcome compared to patients with low RDW (152 (14.3%) vs. 16 (1.4%); p < 0.001). RDW was found to be a strong predictor for mortality in unadjusted model (OR 1.32; 95% CI 1.27–1.39; p < 0.001). Also, when we adjusted the model for gender, age, comorbidities and main diagnosis, the association of RDW and outcome remained significant (**Table 2**). In a fully adjusted model, however, including age, gender, and the various laboratory parameters, there was no significant association of RDW and outcome (OR 1.02 (95% CI 0.93–1.12).

**Table 2:** Associations of RDW adjusted for adverse medical outcomes.

	*Mortality*		*ICU admission*		*Readmission*	
	OR (95%CI)	*p value*	OR (95%CI)	*p value*	OR (95%CI)	*p value*
*Models including clinical information readily available at ED admission*						
Unadjusted RDW model	1.32 (1.27, 1.39)	*<0*.*001*	1.08 (1.02, 1.16)	*0*.*013*	1.11 (1.06, 1.17)	*<0*.*001*
Model 1	1.27 (1.21, 1.33)	*<0*.*001*	1.07 (1.00, 1.14)	*0*.*068*	1.11 (1.05, 1.16)	*<0*.*001*
Model 2	1.20 (1.14, 1.27)	*<0*.*001*	1.08 (1.00, 1.16)	*0*.*028*	1.08 (1.03, 1.14)	*0*.*004*
Model 3	1.19 (1.12, 1.25)	*<0*.*001*	1.06 (1.00, 1.15)	*0*.*093*	1.07 (1.02. 1.14)	*0*.*011*
Model 4	1.07 (0.98, 1.17)	*0*.*131*	0.97 (0.85, 1.10)	*0*.*588*	1.03 (0.93, 1.16)	*0*.*489*
Full model	1.02 (0.93, 1.12)	*0*.*664*	0.97 (0.84, 1.12)	*0*.*679*	1.02 (0.92, 1.15)	*0*.*676*

Model 1: RDW, age, gender. Model 2: RDW, age, gender, comorbidities. Model 3: RDW, age, gender, comorbidities, main diagnosis. Model 4: RDW, age, gender, laboratory parameters. Full model: RDW, age, gender, comorbidities, main diagnosis, laboratory parameters. OD, odds ratios; 95%CI, relative 95% confidence intervals

### Association of RDW and different biological pathways

To better understand associations of RDW with different biological pathways, we used univariate linear regression analysis for all variables included in the full model and report coefficient of determination (R^2^) (**Table 3**). Overall, the adjusted R^2^ of all parameters included in the full model was 0.42 suggesting that 42% of variation in RDW is explained by these pathways. When looking at single parameters, hemoglobin and proADM had highest correlation with RDW as expressed by adjusted R^2^ of 0.26 and 0.24, respectively. Since there is a physiological influence of hemoglobin on RDW, we also calculated an analysis without including hemoglobin. This model still had a high coefficient of determination of 0.37. Overall, these analysis suggested strong correlations of RDW with different pathways, particularly inflammation (R^2^ = 0.30; p<0.001), nutrition (R^2^ = 0.20; p<0.001) and blood diseases (R^2^ = 0.30; p<0.001).

**Table 3:** Association of RDW with different parameters and biological pathways.

	β-Coefficient	R^2^	*p-value*
**Full model**	**-**	**0.42**	***< 0*.*001***
**Full model without hemoglobin**	**-**	**0.37**	***< 0*.*001***
**Full model without proADM**	**-**	**0.38**	***< 0*.*001***
**Model for inflammation**	**-**	**0.30**	***< 0*.*001***
Pro-adrenomedullin	0.10 (0.09; 0.10)	0.24	*< 0*.*001*
Copeptin	0.02 (0.02; 0.03)	0.07	*< 0*.*001*
Procalcitonin	0.01 (0.01; 0.02)	0.02	*< 0*.*001*
C-reactive protein	0.02 (0.02; 0.03)	0.04	*< 0*.*001*
Albumin	-0.01 (-0.01; -0.009)	0.21	*< 0*.*001*
White blood cell count	0.02 (0.01; 0.02)	0.01	*< 0*.*001*
Absolute neutrophil count	0.005 (0.004; 0.006)	0.03	*< 0*.*001*
Infectious and parasitic diseases	0.01 (-0.003; 0.2)	0.0005	*0*.*144*
**Model for blood**	**-**	**0.30**	***< 0*.*001***
INR	0.05 (0.048; 0.06)	0.07	*< 0*.*001*
Platelets	0.0001 (0.00004; 0.0001)	0.003	*< 0*.*001*
Hemoglobin	-0.0029 (-0.003; -0.0027)	0.26	*< 0*.*001*
Mean corpuscular volume (MCV)	-0.001 (-0.001; 0.0001)	0.0002	*0*.*973*
Diseases of the blood	0.11 (0.098; 0.131)	0.04	*< 0*.*001*
**Model for kidneys**	**-**	**0.07**	***< 0*.*001***
Creatinine	-1.46 (-1.65; -1.27)	0.05	*< 0*.*001*
Comorbidity renal failure	0.083 (0.078; 0.099)	0.06	*< 0*.*001*
**Model for nutrition**	**-**	**0.20**	***< 0*.*001***
Glucose	0.042 (0.03; 0.055)	0.01	*< 0*.*001*
Calcium	-0.142 (-0.171; -0.144)	0.02	*< 0*.*001*
Corrected calcium	-1.75 (-1.92; -1.57)	0.08	*< 0*.*001*
Comorbidity diabetes	0.0403 (0.029; 0.051)	0.01	*< 0*.*001*
Endocrine and metabolic diseases	0.004 (-0.027; 0.035)	0	*0*.*811*
**Model for tumor**	**-**	**0.09**	***< 0*.*001***
Neoplastic diseases	0.115 (0.098; 0.131)	0.04	*< 0*.*001*
Comorbidity tumor	0.106 (0.096; 0.116)	0.09	*< 0*.*001*
**Model for cardiovascular system**	**-**	**0.02**	***< 0*.*001***
Diseases of the circulatory system	-0.0014 (-0.01; 0.007)	0	*0*.*747*
Comorbidity hypertension	0.036 (0.028; 0.043)	0.02	*< 0*.*001*
Comorbidity coronary heart disease	0.011 (-0.0008; 0.022)	0.0008	*0*.*067*
**Model for lungs**	**-**	**0.01**	***< 0*.*001***
Diseases of the respiratory system	0.029 (0.014; 0.045)	0.003	*< 0*.*001*
Comorbidity COPD	0.069 (0.052; 0.087)	0.01	*< 0*.*001*

Laboratory parameters were transformed to reach normal distribution before entering into the statistical models.

**Fig 1:**
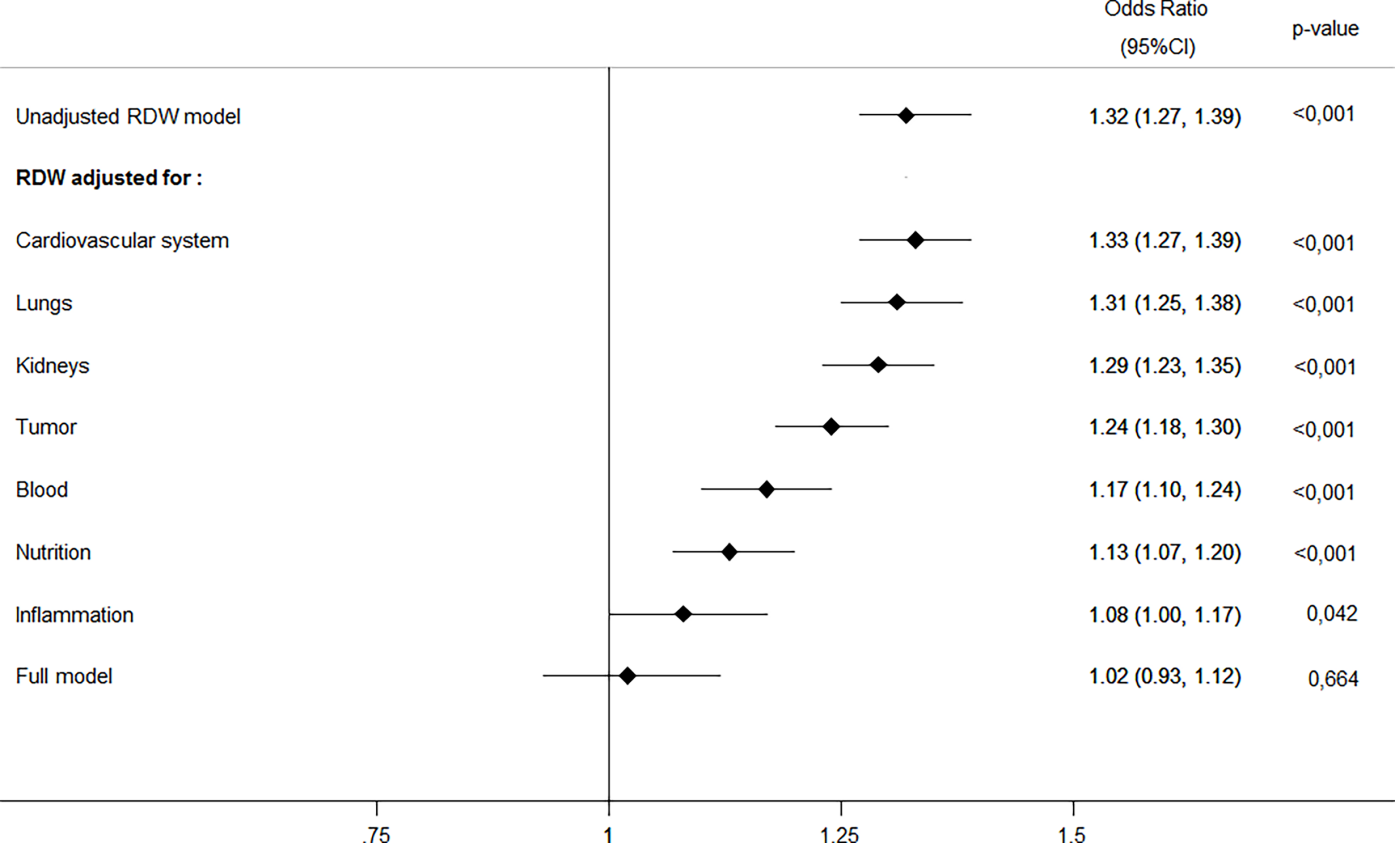
Predictive value of RDW adjusted for different pathophysiological pathways and full model.

In a next step, we adjusted our main mortality model for different biological pathways including factors from the cardiovascular system, lungs, kidneys, tumor, blood, nutrition and inflammation. As shown in **Table 4**and **Fig 1**, the association of RDW and mortality was reduced after addition of these pathways into the statistical model. This suggests that some of the association of RDW and mortality found in univariate analysis is explained by these pathways, particularly inflammation, nutrition and blood diseases.

**Table 4:** Associations of RDW adjusted for different biological pathways.

	*Mortality*		*ICU admission*		*Readmission*	
	OR (95%CI)	*p value*	OR (95%CI)	*p value*	OR (95%CI)	*p value*
*Models including clinical information readily available at ED admission*						
Unadjusted RDW model	1.32 (1.27, 1.39)	*<0*.*001*	1.08 (1.02, 1.16)	*0*.*013*	1.11 (1.06, 1.17)	*<0*.*001*
**RDW adjusted for:**						
Cardiovascular system	1.33 (1.27, 1.39)	*<0*.*001*	1.09 (1.02, 1.16)	*0*.*011*	1.11 (1.05, 1.17)	*<0*.*001*
Lungs	1.31 (1.25, 1.38)	*<0*.*001*	1.07 (1.01, 1.15)	*0*.*025*	1.11 (1.06, 1.17)	*<0*.*001*
Kidneys	1.29 (1.23, 1.35)	*<0*.*001*	1.04 (0.97, 1.17)	*0*.*266*	1.11 (1.06, 1.18)	*<0*.*001*
Tumor	1.24 (1.18, 1.30)	*<0*.*001*	1.09 (1.03, 1.17)	*0*.*005*	1.08 (1.03, 1.14)	*0*.*003*
Blood	1.17 (1.10, 1.24)	*<0*.*001*	1.08 (1.01, 1.17)	*0*.*035*	1.09 (1.03, 1.16)	*0*.*004*
Nutrition	1.13 (1.07, 1.20)	*<0*.*001*	0.97 (0.90, 1.06)	*0*.*591*	1.10 (1.04, 1.16)	*0*.*001*
Inflammation	1.08 (1.00, 1.17)	*0*.*042*	0.97 (0.86, 1.09)	*0*.*587*	1.01 (0.91, 1.12)	*0*.*844*

Cardiovascular system: RDW, diseases of the circulatory system, comorbidities: hypertension, coronary heart disease. Lungs: RDW, diseases of the respiratory system, comorbidity: chronic obstructive pulmonary disease. Kidneys: RDW, comorbidity: renal failure, creatinine. Tumor: RDW, neoplastic diseases, comorbidity: tumor. Blood: RDW, INR, Platelets count, hemoglobin, mean corpuscular volume, diseases of the blood and blood-forming organs. Nutrition: RDW, glucose, calcium, calcium corrected, endocrine and metabolic diseases, comorbidity: diabetes. Inflammation: RDW, pro-adrenomedullin, copeptin, procalcitonin, C-reactive protein, albumin, leukocytes, absolute neutrophil count, infectious and parasitic diseases. OD, odds ratios; 95%CI, relative 95% confidence intervals. Laboratory parameters were transformed to reach normal distribution before entering into the statistical models.

### Subgroup analysis for anemia

We also performed a subgroup analysis in patients with anemia. We divided the population by hemoglobin level, considering patients with hemoglobin <120 g/l as anemic. RDW had a stronger association with outcome in the patient group with no anemia (OR 1.42; 95% CI 1.3–1.55 vs. 1.13; 95% CI 1.07–1.20) compared to patients with anemia. Details of the subgroup analysis are presented in the **S1 Table, S2 Table**).

### Secondary endpoints

Similar to the mortality endpoint, RDW was also associated with secondary endpoints in unadjusted model analysis, namely unplanned hospital readmissions (OR 1.11; 95% CI 1.06–1.17; p < 0.001) and ICU admission (OR 1.08; 95% CI 1.02–1.16; p = 0.013). When adjusted for comorbidities, main diagnosis, laboratory findings, there was no more association between RDW and outcomes (**Table 2**).

## Discussion

We investigated RDW as a predictor for 30 days all-cause mortality in a large and well defined cohort of unselected medical patients with parallel measurement of a broad spectrum of laboratory findings and other parameters of different biological pathways. We found RDW to be strongly associated with mortality in unadjusted analysis and also to be correlated to different of these pathways. After step-wise adjustment of the regression models, all of the association was lost suggesting that chronic inflammation, nutrition and blood disease, among other biological pathways, are the main factors explaining the association of RDW and mortality. Thus, because erythrocytes circulate for around 120 days and erythropoiesis is affected by many chronic disease factors including inflammation, kidney diseases, malignancies, autoimmune diseases, chronic rejection after solid-organ transplantation [14], as well as oxidative stress [15], our data suggest that RDW mirrors chronic disease and may thus be viewed as a unspecific but outcome-relevant “chronic disease marker”.

Regarding the association of RDW and inflammation, the findings of this study are in line with previous research reporting similar associations in distinct patient populations. RDW was found to be closely associated with different acute-phase inflammatory markers in different studies. For example, IL-6, TNF-RI and TNF-RII were significantly higher in the highest RDW patients with heart failure [16]. RDW >14% was correlated with significantly higher copeptin, mid-regional pro-adrenomedulin and soluble urokinase plasminogen activating receptor levels in patients admitted to the ED with acute infections in another study [17]. Also Lippi [18] found strong association of RDW with hsCRP and ESR independent of other confounding factors in a large cohort of unselected outpatients. Herein, our study confirms these findings and furthers broadens these results to unselected medical inpatients. Also, we included novel markers such as pro-adrenomedullin, copeptin and/or procalcitonin.

Regarding other biological pathways, associations between RDW and oxidative stress has been reported. The Women’s Health and Aging Study I found that serum selenium was an independent predictor of RDW and could potentially moderate effects on RDW through IL-6 [19]. Meanwhile it is known, that selenium deficiency decreases glutathione peroxidase (GSH-Px) activity in erythrocytes, while GSH-Px is an important antioxidant enzyme contained in erythrocytes [20,21]. The erythrocyte morphology could be linked to oxidative stress and chronic inflammation in patients with metabolic syndrome [15]. All those studies are also suggesting that there is strong correlation between RDW and inflammatory and oxidative processes.

Additionally there are findings suggesting that RDW is related to the aging process. Studies about age-related clonal hematopoiesis revealed that subjects with RDW >14.5% had significantly more somatic mutations in peripheral-blood cells [22]. In a large multi-ethnic population based study investigating telomere lengths of genomic DNA isolated from circulating leukocytes identified that shorter telomere length is strongly associated with greater RDW [23]. Taken these findings together, RDW may be viewed as a marker mirroring the ageing process in the body.

Bion [24] argued that one of the cornerstones in interpreting risk and outcome together with response and therapy is the “physiologic reserve” of the body. The concept of “physiologic reserve”, mainly used in intensive care units, is defined as the critical threshold of physiologic adaptation that an individual can mount in response to acute illness before its failure to adapt predisposes to homeostatic failure and decompensation [25]. Goffaux [26] described it as the outcome of the interaction of aging, lifestyle, and disease. Meanwhile Bion [24], for example, developed an integrated model taking into consideration genetic factors, cardiovascular health, oxygen delivery, immune state and nutrition. One could speculate that RDW could be a “physiologic reserve” marker mirroring different biological pathways including inflammation, nutritional status, disease of blood forming organs, immune system, oxidative stress [15,19] and aging [23,26]. This is also supported by the fact that addition of RDW improved different disease scores scores [10–12].

Because of the influence of anemia on RDW, we performed subgroup analyses in patients with and without anemia. While results were significant in both groups, the subgroup of non-anemic patients displayed stronger effects of RDW and outcome. This suggests that clinical interpretation of RDW should be in conjunction with hemoglobin level.

The following strength and limitations of this study should be noted. This is a large-scale, prospective study that measured RDW in parrallel to other parameters and markers of disease and was thus able to adjust the regression models by a variety of biological pathways. Nevertheless, our study has some limitations. First, this study was a single-centre study including Swiss patients and results may not be applicable to other populations. Second, this was an observational study. Third, 30-day mortality was obtained by direct contact, since standardized surveillance methods were not available. Thus, loss to follow up or mis-characterization of outcomes is possible because death records were not used. Finally, data about patients receiving transfusions or anaemia cause (including iron status, vitamin B12 level) were not available.

## Conclusion

As a surrogate combining different biological pathways such as chronic inflammation, malnutrition and blood disease, RDW may be viewed as a general “chronic disease and co-morbidity marker” and a strong but not independent surrogate marker for mortality in unselected medical patients being admitted to the inpatient ward through the ED.

## Supporting information

S1 Table Associations of RDW adjusted for different biological pathways by patients without anemia. Cardiovascular system: RDW, diseases of the circulatory system, comorbidities: hypertension, coronary heart disease.
Lungs: RDW, diseases of the respiratory system, comorbidity: chronic obstructive pulmonary disease.
Kidneys: RDW, comorbidity: renal failure, creatinine.
Tumor: RDW, neoplastic diseases, comorbidity: tumor.
Blood: RDW, INR, Platelets count, hemoglobin, mean corpuscular volume, diseases of the blood and blood-forming organs.
Nutrition: RDW, glucose, calcium, calcium corrected, endocrine and metabolic diseases, comorbidity: diabetes.
Inflammation: RDW, pro-adrenomedullin, copeptin, procalcitonin, C-reactive protein, albumin, leukocytes, absolute neutrophil count, infectious and parasitic diseases.
OD, odds ratios; 95%CI, relative 95% confidence intervals.
Laboratory parameters were transformed to reach normal distribution before entering into the statistical models.
(DOCX)

S2 Table Associations of RDW adjusted for different pathophysiological pathways by patients with anemia. Cardiovascular system: RDW, diseases of the circulatory system, comorbidities: hypertension, coronary heart disease.
Lungs: RDW, diseases of the respiratory system, comorbidity: chronic obstructive pulmonary disease.
Kidneys: RDW, comorbidity: renal failure, creatinine.
Tumor: RDW, neoplastic diseases, comorbidity: tumor.
Blood: RDW, INR, Platelets count, hemoglobin, mean corpuscular volume, diseases of the blood and blood-forming organs.
Nutrition: RDW, glucose, calcium, calcium corrected, endocrine and metabolic diseases, comorbidity: diabetes.
Inflammation: RDW, pro-adrenomedullin, copeptin, procalcitonin, C-reactive protein, albumin, leukocytes, absolute neutrophil count, infectious and parasitic diseases.
OD, odds ratios; 95%CI, relative 95% confidence intervals.
Laboratory parameters were transformed to reach normal distribution before entering into the statistical models.
(DOCX)

S1 Dataset Open dataset of this study in accordance with data availability policy. (XLSX)

## Abbreviations

ANC: absolute neutrophil count
CBC: complete blood count
COPD: chronic obstructive pulmonary disease
CRP: C-reactive protein
ED: emergency department
GSH-Px: glutathione peroxidase
hsCRP: high sensitive C-reactive protein
ICD-10: International Classification of Diseases (10th Revision)
ICU: intensive care unit
IL-6: interleukin 6
MCV: mean corpuscular volume
OD: odds ratios
PCT: Procalcitonin
ProADM: Pro-adrenomedullin
RDW: red cell distribution width
TNF-RI: tumor necrosis factor receptor 1
TNF-RII: tumor necrosis factor receptor 2
WBC: white blood cell count
