# Supplementary material for: Biological pathways underlying the association of red cell distribution width and adverse clinical outcome: Results of a prospective cohort study
Source: PLoS One. 2018 Jan 17;13(1):e0191280. doi: 10.1371/journal.pone.0191280 (PMC5771602; doi:10.1371/journal.pone.0191280)
Supplement: S1 Table — Cardiovascular system: RDW, diseases of the circulatory system, comorbidities: hypertension, coronary heart disease. Lungs: RDW, diseases of the respiratory system, comorbidity: chronic obstructive pulmonary disease. Kidneys: RDW, comorbidity: renal failure, creatinine. Tumor: RDW, neoplastic diseases, comorbidity: tumor. Blood: RDW, INR, Platelets count, hemoglobin, mean corpuscular volume, diseases of the blood and blood-forming organs. Nutrition: RDW, glucose, calcium, calcium corrected, endocrine and metabolic diseases, comorbidity: diabetes. Inflammation: RDW, pro-adrenomedullin, copeptin, procalcitonin, C-reactive protein, albumin, leukocytes, absolute neutrophil count, infectious and parasitic diseases. OD, odds ratios; 95%CI, relative 95% confidence intervals. Laboratory parameters were transformed to reach normal distribution before entering into the statistical models. (DOCX) [file pone.0191280.s001.docx]

**S1 Table. Associations of RDW adjusted for different** [**biological**](https://www.google.ch/search?biw=2021&bih=1005&q=pathophysiological&spell=1&sa=X&ved=0ahUKEwjh3pnCvuTQAhUB1xoKHdOHCZwQvwUIFygA) **pathways by patients without anemia**

|  | ***Mortality*** |  | ***ICU admission*** |  | ***Readmission*** |  |
| --- | --- | --- | --- | --- | --- | --- |
|  | OR (95%CI) | *p value* | OR (95%CI) | *p value* | OR (95%CI) | *p value* |
| *Models including clinical information readily available at ED admission* | | | | | | |
| Unadjusted RDW model | 1.42 (1.30, 1.55) | *<0.001* | 1.17 (1.06, 1.29) | *0.001* | 1.16 (1.07, 1.26) | *<0.001* |
| **RDW adjusted for :** | | | | | | |
| Cardiovascular system | 1.40 (1.28, 1.54) | *<0.001* | 1.16 (1.05, 1.29) | *0.005* | 1.16 (1.06, 1.26) | *<0.001* |
| Lungs | 1.39 (1.27, 1.52) | *<0.001* | 1.16 (1.04, 1.28) | *0.005* | 1.15 (1.06, 1.25) | *<0.001* |
| Kidneys | 1.37 (1.25, 1.50) | *<0.001* | 1.11 (0.99, 1.24) | *0.058* | 1.16 (1.07, 1.27) | *<0.001* |
| Tumor | 1.36 (1.24, 1.49) | *<0.001* | 1.16 (1.06, 1.29) | *0.002* | 1.13 (1.04,1.23) | *0.004* |
| Blood | 1.35 (1.22, 1.50) | *<0.001* | 1.18 (1.06, 1.31) | *0.003* | 1.13 (1.03, 1.23) | *0.008* |
| Nutrition | 1.21 (1.09, 1.35) | *<0.001* | 1.05 (0.93, 1.17) | *0.446* | 1.12 (1.03, 1.22) | *0.011* |
| Inflammation | 1.03 (0.83, 1.24) | *0.742* | 0.97 (0.81, 1.18) | *0.822* | 1.09 (0.94, 1.29) | *0.251* |
|  | | | | | | |

*Cardiovascular system: RDW, diseases of the circulatory system, comorbidities: hypertension, coronary heart disease*

*Lungs: RDW, diseases of the respiratory system, comorbidity: chronic obstructive pulmonary disease*

*Kidneys: RDW, comorbidity: renal failure, creatinine*

*Tumor: RDW, neoplastic diseases, comorbidity: tumor*

*Blood: RDW, INR, Platelets count, hemoglobin, mean corpuscular volume, diseases of the blood and blood-forming organs*

*Nutrition: RDW, glucose, calcium, calcium corrected, endocrine and metabolic diseases, comorbidity: diabetes*

*Inflammation: RDW, pro-adrenomedullin, copeptin, procalcitonin, C-reactive protein, albumin, leukocytes, absolute neutrophil count, infectious and parasitic diseases*

*OD, odds ratios; 95%CI, relative 95% confidence intervals*

*Laboratory parameters were transformed to reach normal distribution before entering into the statistical models.*
