# Supplementary material for: Biological pathways underlying the association of red cell distribution width and adverse clinical outcome: Results of a prospective cohort study
Source: PLoS One. 2018 Jan 17;13(1):e0191280. doi: 10.1371/journal.pone.0191280 (PMC5771602; doi:10.1371/journal.pone.0191280)
Supplement: S2 Table — Cardiovascular system: RDW, diseases of the circulatory system, comorbidities: hypertension, coronary heart disease. Lungs: RDW, diseases of the respiratory system, comorbidity: chronic obstructive pulmonary disease. Kidneys: RDW, comorbidity: renal failure, creatinine. Tumor: RDW, neoplastic diseases, comorbidity: tumor. Blood: RDW, INR, Platelets count, hemoglobin, mean corpuscular volume, diseases of the blood and blood-forming organs. Nutrition: RDW, glucose, calcium, calcium corrected, endocrine and metabolic diseases, comorbidity: diabetes. Inflammation: RDW, pro-adrenomedullin, copeptin, procalcitonin, C-reactive protein, albumin, leukocytes, absolute neutrophil count, infectious and parasitic diseases. OD, odds ratios; 95%CI, relative 95% confidence intervals. Laboratory parameters were transformed to reach normal distribution before entering into the statistical models. (DOCX) [file pone.0191280.s002.docx]

**S2 Table. Associations of RDW adjusted for different** [**pathophysiological**](https://www.google.ch/search?biw=2021&bih=1005&q=pathophysiological&spell=1&sa=X&ved=0ahUKEwjh3pnCvuTQAhUB1xoKHdOHCZwQvwUIFygA) **pathways by patients with anemia**

|  | ***Mortality*** |  | ***ICU admission*** |  | ***Readmission*** |  |
| --- | --- | --- | --- | --- | --- | --- |
|  | OR (95%CI) | *p value* | OR (95%CI) | *p value* | OR (95%CI) | *p value* |
| *Models including clinical information readily available at ED admission* | | | | | | |
| Unadjusted RDW model | 1.13 (1.07, 1.20) | *<0.001* | 1.01 (0.91, 1.13) | *0.807* | 1.07 (0.99, 1.16) | *0.085* |
| **RDW adjusted for :** | | | | | | |
| Cardiovascular system | 1.13 (1.07, 1.20) | *<0.001* | 1.02 (0.91, 1.13) | *0.753* | 1.07 (0.99, 1.16) | *0.081* |
| Lungs | 1.13 (1.06, 1.20) | *<0.001* | 1.02 (0.91, 1.13) | *0.783* | 1.08 (0.99, 1.16) | *0.066* |
| Kidneys | 1.13 (1.07, 1.20) | *<0.001* | 1.01 (0.90, 1.12) | *0.891* | 1.07 (0.99, 1.16) | *0.077* |
| Tumor | 1.09 (1.02, 1.16) | *0.014* | 1.05 (0.94, 1.16) | *0.390* | 1.06 (0.98, 1.15) | *0.149* |
| Blood | 1.09 (1.01, 1.16) | *0.020* | 1.00 (0.89, 1.14) | *0.895* | 1.06 (0.98, 1.16) | *0.165* |
| Nutrition | 1.08 (1.00, 1.15) | *0.036* | 0.99 (0.88, 1.12) | *0.884* | 1.09 (1.00, 1.17) | *0.039* |
| Inflammation | 1.08 (0.99, 1.18) | *0.842* | 1.11 (0.95, 1.30) | *0.198* | 1.02 (0.87, 1.18) | *0.842* |

*Cardiovascular system: RDW, diseases of the circulatory system, comorbidities: hypertension, coronary heart disease*

*Lungs: RDW, diseases of the respiratory system, comorbidity: chronic obstructive pulmonary disease*

*Kidneys: RDW, comorbidity: renal failure, creatinine*

*Tumor: RDW, neoplastic diseases, comorbidity: tumor*

*Blood: RDW, INR, Platelets count, hemoglobin, mean corpuscular volume, diseases of the blood and blood-forming organs*

*Nutrition: RDW, glucose, calcium, calcium corrected, endocrine and metabolic diseases, comorbidity: diabetes*

*Inflammation: RDW, pro-adrenomedullin, copeptin, procalcitonin, C-reactive protein, albumin, leukocytes, absolute neutrophil count, infectious and parasitic diseases*

*OD, odds ratios; 95%CI, relative 95% confidence intervals*

*Laboratory parameters were transformed to reach normal distribution before entering into the statistical models.*
